# Vaccine Champions Training Program: Empowering Community Leaders to Advocate for COVID-19 Vaccines

**DOI:** 10.3390/vaccines10111893

**Published:** 2022-11-09

**Authors:** Jessica Kaufman, Isabella Overmars, Julie Leask, Holly Seale, Melanie Chisholm, Jade Hart, Kylie Jenkins, Margie Danchin

**Affiliations:** 1 Vaccine Uptake Group, Murdoch Children’s Research Institute, Parkville, VIC 3052, Australia; 2 Department of Paediatrics, The University of Melbourne, Parkville, VIC 3010, Australia; 3 Susan Wakil School of Nursing and Midwifery, University of Sydney, Camperdown, NSW 2006, Australia; 4 School of Population Health, University of New South Wales, Kensington, NSW 1466, Australia; 5 Victorian Department of Health, Melbourne, VIC 3000, Australia; 6 School of Population and Global Health, The University of Melbourne, Carlton, VIC 3010, Australia; 7 The Royal Children’s Hospital, Parkville, VIC 3052, Australia

**Keywords:** vaccination, COVID-19 vaccine, community engagement, communication, peer-to-peer

## Abstract

Strong community engagement has been critical to support COVID-19 vaccine uptake in Australia and elsewhere. Community engagement builds trust, enables tailored information dissemination and shapes social norms. Engagement is particularly important in communities with greater vaccine hesitancy, lower health literacy and mistrust in authorities. Early in 2021, as a team of vaccine social scientists and clinicians, we developed a program to train and empower community, faith, industry and healthcare leaders to advocate for COVID-19 vaccines as “vaccine champions”. We partnered with the Victorian Department of Health to deliver 91 online Vaccine Champions sessions from March 2021 to June 2022. Over 80 people who received this training were supported by the Department of Health to become formal vaccine champions, independently delivering over 100 locally tailored information sessions. Our survey evaluation of 20 sessions delivered in 2022 found most participants (94%, 118/125) felt more confident to discuss safety and effectiveness of COVID-19 vaccines and find relevant information after attending a session. We also recorded >90% participant satisfaction with training content, format and presentation. Qualitative feedback from two group interviews highlighted the value of vaccine communication role plays and opportunities for discussion. In this brief report, we present an overview of the Vaccine Champions program, evaluation and next steps.

## 1. Introduction

While Australia has achieved very high COVID-19 vaccine coverage among eligible adults, targeted efforts and unprecedented co-ordination between state and Commonwealth governments were required to overcome challenges. Research from early in the pandemic identified certain groups with lower intention to receive a COVID-19 vaccine, including people from culturally and linguistically diverse (CALD) backgrounds, people living in remote or regional areas, Aboriginal and Torres Strait Islander people and people with a disability [1,2,3,4,5]. The reasons for reduced intention and uptake include both practical access barriers and acceptance issues [4,5,6].

Communities at highest risk of experiencing the greatest burden of disease from COVID-19 are often those with lower levels of health literacy or numeracy and those who speak a language other English [7,8]. For example, by June 2022, those whose country of birth was in the Middle East had the highest age-standardized death rate at 46.9 deaths per 100,000 people, compared with those whose country of birth was Australia, at 7.6 deaths per 100,000 [9]. While there are a range of reasons for this, a key contributor is lower vaccine uptake due to personal or cultural experiences or beliefs, mistrust in government or medical professionals, or lack of appropriate and accessible information [10]. Messages shared by an external figure like a government spokesperson may be less accessed or trusted and therefore have less impact than messages shared by a known individual from within a particular community. Hence, it is important to empower different types of people from different communities to advocate for vaccination as vaccine champions [11].

A vaccine champion is someone who has a trusted position in a community or workplace and is passionate about encouraging people to receive vaccines. Anyone can be a vaccine champion—they do not require academic or clinical qualifications. Vaccine champions have the most impact when they are advocating for vaccination within a community, workplace or setting where they already have established trusted relationships. Some of the key features of an effective vaccine champion are listening to people’s questions and concerns with an open mind; knowing where and how to find reputable information about vaccines; and sharing personal vaccination experiences. Community leaders and members of the public have been used as vaccine champions in many countries, including Nigeria, the UK, US and Australia [12,13,14,15]. Healthcare workers have knowledge and pre-existing skills that make them strong clinical vaccine champions to support vaccination in health settings [16,17]. Champions can also come from within workplaces or faith organizations [18].

Vaccine champions work by shaping social norms and capitalizing on trusted relationships and roles. Vaccination decision-making is influenced by social norms—what people think others are doing, and what they think trusted or influential people want them to do [19]. We are more likely to trust people who look like us [20] or who we feel represent us and our communities. Trust is built over time through transparency, fairness, actions that demonstrate goodwill and shared values.

In this article, we describe the content and implementation of the Vaccine Champions training program in Australia. We assess its impact in a snapshot evaluation and describe the program’s adaptation for countries in the IndoPacific.

### Overview of the Vaccine Champions Program

At the start of the COVID-19 vaccine rollout in February 2021 in Australia, a team of clinical and social science experts from the Collaboration on Social Science and Immunisation (COSSI) (cossi.org.au) developed a program to train and empower vaccine champions, in partnership with the Victorian Department of Health. Drawing on social and behavioral science and vaccine communication evidence and strategies [16,21,22], the Vaccine Champions program aimed to provide education on vaccine development, safety and effectiveness; acknowledge and address community concerns; and engage a range of key leaders to advocate for vaccines. Sessions were designed to reflect health literacy principles including avoiding jargon, using pictures and graphical representations of complex information, focusing on action-oriented recommendations, demonstration, checking comprehension and encouraging an open forum for discussion and questions [23].

**Figure 1:**
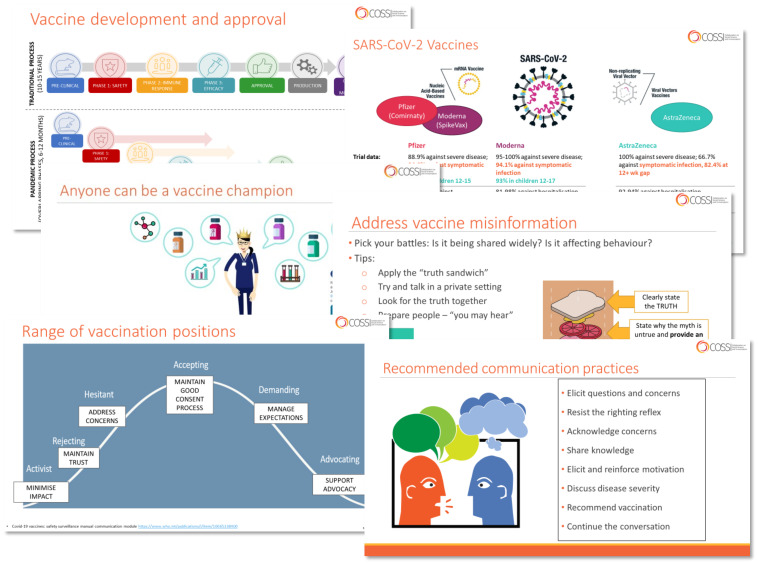
Sample slides from Vaccine Champions training modules.

The Vaccine Champions program was designed for delivery face-to-face or online via Zoom or Microsoft Teams, to maximize reach during COVID-19 lockdowns. The program was delivered between March 2021 and June 2022. Each standalone training session comprised a 60–90 min webinar using PowerPoint slides (Figure 1), scripted role play demonstrations and interactive question and answer time. The sessions were delivered by one or two presenters with expertise in vaccines, pediatrics, infectious diseases, communication and social and behavioral science. The Victorian Department of Health engagement and communications teams organized and promoted sessions across governmental portfolios and through industry and community networks.

The sessions began with a vaccine information module that lasted about 40 min and presented up-to-date data on COVID-19 vaccine development and testing, safety, side effects, effectiveness and vaccine eligibility, which was subject to change. The content was updated weekly to reflect emerging safety and real-world effectiveness data and recommendations from the Australian Technical Advisory Group on Immunisation (ATAGI), the Therapeutic Goods Administration (TGA), related bodies overseas and academic literature. It covered adult and child COVID-19 vaccines as they became available. After this module, audience members were encouraged to ask any questions they had or share their experiences or concerns. The second module on vaccine advocacy explained risk communication principles and provided guidance on addressing misinformation by debunking or ‘pre-bunking’ common myths. Participants learned practical strategies to be active vaccine champions in their workplace or community, such as sharing their personal vaccination experiences and helping others find reliable information. The third module on vaccine communication focused on evidence-based communication practices to engage effectively with people who are vaccine hesitant in person, in online forums, through social media and in healthcare environments. The presenters used scripted role plays to demonstrate ineffective and effective conversations and invited reflection and feedback. Another Q&A period followed the final module.

From March 2021 to June 2022, 91 sessions were delivered to different groups in Australia. Most sessions averaged between 25 and 40 participants (range 6 to 2000+), with higher participation in 2021 than 2022. Audiences included healthcare workers, those working with CALD communities including community leaders, migration settlement service staff, and bilingual workers, people working with those who are homeless or who have disabilities, Aboriginal health groups, teachers and representatives from the Department of Education, people working in the emergency management sector or transport industry, police, trade union representatives, faith leaders, youth organizations, LGBTQIA+ organizations, sporting bodies, and the general community.

Content and strategies were tailored to each audience as appropriate. Most sessions were delivered during the workday, but some were scheduled in the early morning or evening to accommodate diverse schedules. Some sessions were co-facilitated with Aboriginal health experts and one session was delivered on Facebook live with asynchronous translation into Arabic.

More than 80 people who received the training went on to become formal vaccine champions, supported by the Victorian Department of Health to deliver their own information sessions and encourage vaccine uptake in their own unique contexts. They were provided with adapted versions of the training materials and delivered more than 100 sessions within their communities. These formal champions attended regular sessions to provide them with the most up-to-date data to share.

## 2. Materials and Methods

### 2.1. Study Design

We evaluated a sub-set of twenty sessions delivered in Victoria in 2022, using a mixed methods approach involving surveys and group interviews.

### 2.2. Participants and Recruitment

All participants who attended one of the online Vaccine Champions training program sessions between January and June 2022 were invited to complete a post-training evaluation survey. The link was provided in the webinar chat and was emailed to participants again after the session. Consent was implied by survey completion. Participants also had the option of providing their contact details if they were willing to take part in an evaluation interview.

Participants who completed the evaluation survey and expressed interest in an interview were invited using email to take part in a group interview. The Victorian Department of Health also emailed the link to formal vaccine champions trained in 2021 and invited them to participate. Interested participants contacted the study team, provided informed consent, and were enrolled. Participants were given a $50 voucher for their time. Ethics approval was received from the Royal Children’s Hospital Human Research Ethics Committee (HREC/82021/RCHM-2022-300421).

### 2.3. Data Collection

Participant confidence and satisfaction with the program were assessed through an online REDCap survey. The survey included questions about participants’ demographics (identity of group/network), evaluation of the training sessions (satisfaction with content about vaccine safety and effectiveness, strategies for discussing vaccines, role plays, question and answer section, and quality of presenters), and impact of the training (changes in confidence to talk about risks and benefits of, find appropriate resources on, and initiate a conversation with a hesitant person about COVID-19 vaccines) (Table A1).

Two semi-structured group interviews were conducted to gather more detailed feedback on the content and format of the training and to explore the experiences of vaccine champions, particularly those who went on deliver their own sessions. Interviews were conducted by one researcher (IO) with a second researcher present as an observer and note-taker. Interviews were held between May–June 2022 using an interview guide (Table A2). They were recorded and transcribed.

### 2.4. Analysis

Survey responses were analyzed using descriptive statistics. Categorical responses are presented as number and percentages. Two authors (I.O. and J.K.) analyzed the group interview transcripts using a deductive framework analysis approach using key topics from the interview guide [24].

## 3. Results

### 3.1. Sample Characteristics

**Figure 2:**
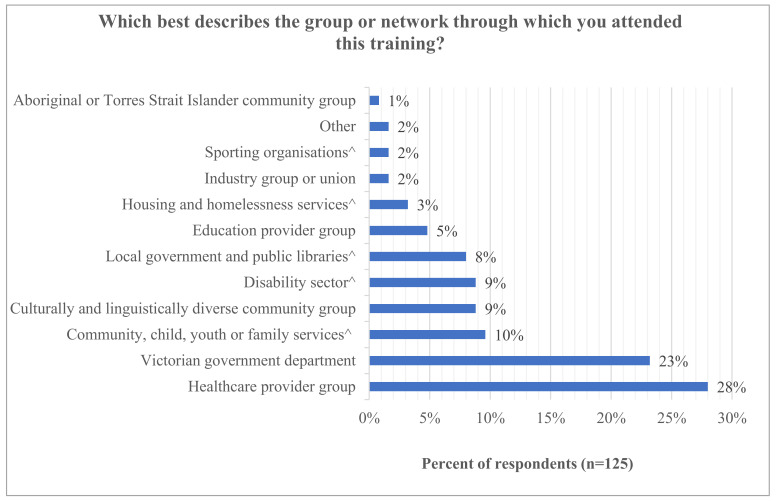
Organizations of session participants. ^ Categories created based on free text responses to “Other” option.

A total of 125 participants provided survey data out of approximately 500 attendees who received a survey link (estimated response rate 25%). Twelve participants took part in the group interviews (10 female). The majority of survey participants who attended the training were from healthcare provider groups (28%; 35/125) and the Victorian Government (23%; 29/125) (Figure 2). Types of participants were largely similar between the 2021 sessions and the evaluated 2022 sessions. However, more of the 2022 sessions were open to the general public, and more were delivered to mixed audiences, rather than targeting a single organization or industry.

Characteristics of the 12 interview participants are reported in Table 1. All interview participants had attended at least one Vaccine Champions training session, and 8 were formal vaccine champions who had delivered their own community or workplace sessions following the training.

### 3.2. Confidence and Satisfaction

Nearly all participants felt more confident in their ability to discuss COVID-19 vaccine risks and benefits and to find trustworthy information (Table 2). Most participants reported that they were likely or very likely to initiate a conversation about COVID-19 vaccines with a hesitant person after attending the training. Most participants were very satisfied with all aspects of the program and felt the session was the right length.

### 3.3. Training Experience and Feedback

#### 3.3.1. Content

Interview participants described the information about vaccines as informative and helpful, adding it was important to help them respond to people’s specific questions. Most participants had attended a training session multiple times as they used it to gain up-to-date information on COVID-19 vaccines: *“I kept attending because I knew that the [training materials] were constantly being updated, and things changed”*. Those from a clinical background emphasized the importance of the up-to-date detailed information on vaccines, but those from a non-clinical background would have preferred simpler information: *“I was a bit confronted by a lot of that medical stuff, and describing the vaccine, and what’s an mRNA…I had a bit of a, ‘I don’t need to know this’”*. Despite this, participants said that the information was *“very digestible”* and delivered in a way they could understand, even if they did not always feel it was relevant to them.

Information provided on how to communicate with a vaccine hesitant person and how to counter misinformation was described as useful and important.

*“The role play was really good… they did a really good job addressing how to be open and have a chat with people, because everyone has different concerns, so I thought the session was quite useful.”*

Participants would have liked more examples of role plays or of how they could counter misinformation. Participants described wanting more information on how to have a conversation with vaccine refusers, as they felt they encountered those types of conversations often and felt unprepared.

Most participants talked about the value of being given the slides after the session, and often referred to the links, graphs, and statistics included to talk to people or use in their own slides or social media:

*“The graph that showed the protection of the booster shot…we used that in our social media for the multicultural communities, it was a really good way for them to understand the impact.”*

#### 3.3.2. Format

Participants thought the format was appropriate including the order of the content, the inclusion of the role play, and the Q&A sessions. Participants liked that the training occurred online as it was convenient and allowed them to connect with others. *“I wouldn’t have been able to get to* [a face-to-face session], *to be honest. I think, yeah, online was good and it kept me connected to other professionals”*. The Q&A component was described as beneficial, and people enjoyed being able to use the chat function to ask questions and hear about current issues and concerns within community. One participant would have liked to be able to ask questions before the session to help frame the information provided in the context of the critical issues they were observing in their community. Participants described wanting access to a recording of the session to reinforce their knowledge and to share with others, but also understood why they could not have one: *“They didn’t want any permanent record, if you know what I mean, because…* [vaccine information] *changed so much.”*.

#### 3.3.3. Experiences of Formal Vaccine Champions Delivering Vaccine Information Sessions

The eight formal vaccine champions spoke of using the skills from the initial training session and described the experience of delivering their own sessions as *“rewarding”*. Hosting formal sessions was considered part of their job for some participants, and for others it was additional. While the Department of Health made staff available to answer questions and provided access to an online portal with updated information, all the formal champions interviewed talked about the preparation time required to deliver sessions. This included updating their slides and tailoring for the specific audience: *“It’s up to me to put all the presentation together…and you’ve got to update the information…it does take a bit of work”.*

Those who conducted sessions outside of their jobs commented that it was challenging to fit in with their busy lives. All formal champions were offered remuneration on enrolment, but most declined. On reflection, some interview participants commented that remuneration would have been beneficial to account for preparation time.

While some hosted online sessions where they delivered information to an audience, most adapted the session to suit their skill set. Adaptions included bringing other experts to co-deliver a session such as a medical professional or a member of the Department of Health. Other adaptations included creating a session in a Q&A format with representatives from the community such as a parent, a person with disability, or a bilingual community member, who could ask questions to an expert or the trained Vaccine Champion. Participants described that the online format was the best way for them to reach their communities and provide recorded material to them afterwards.

One Vaccine Champion described setting up their own session and how they used the recording afterwards to reach a broader audience:

*“There was a GP, a pathologist, and an infectious disease specialist…I ran through as if I’m asking the questions and they would answer…in the South Asian community it was more of a video to be shared through our WhatsApp channels and various social media channels as well.”*

Another Vaccine Champion located in council had initially been setting up appointments one-on-one with parents to address their concerns, then decided to organize an online session with an immunization specialist and a pediatrician to reach more people, however this had low attendance. To adapt, they used the recording to create smaller short videos to reach their community:

*“I think it was probably too long for someone to sit in front of a screen for as well, in the evening. So, we looked at short messaging…we got a lot more engagement with those kinds of things.”*

#### 3.3.4. Experiences of Informal Vaccine Champions in the Community

Four participants were training attendees who did not deliver formal information sessions but instead used their skills informally within their community. The types of roles people described included acting as a spokesperson in their workplace, helping connect colleagues or community members with up-to-date information, using skills to speak with people who are vaccine hesitant in the community or as part of their job (e.g., in health care), and using skills and knowledge to translate information into their community language or simple language.

Participants described the skills they learnt including how to have a conversation with someone who is hesitant, how to counter misinformation and how to help people think about the sources of their information, how to direct people to services or clarify up-to-date information, linking people with information, and how to *“keep the end of the conversation open”*.

Participants talked about wanting a way to connect with other vaccine champions and have a consistent way to communicate with them, such as through creating *“some kind of forum, a safe space to ask questions that were popping up as things were changing”*. They described wanting to be able to share and hear what questions and concerns were coming from their communities, and to share examples of how to answer the questions. Participants wanted the sessions to continue or to have a place where they could meet to receive up-to-date information:

*“I think it’s not done yet, so I think the program needs to continue and have up-to-date information for us to just keep our heads around what’s relevant now.”*

## 4. Discussion

The Vaccine Champions training program disseminated key COVID-19 vaccine information to thousands of people across Victoria, both through participant attendance at training sessions and community attendance at sessions run by formal Champions. Most participants who took part in the evaluation reported that they were very satisfied with the program and felt more confident to discuss COVID-19 vaccines with others. Those who went on to become formal vaccine champions and deliver information sessions to their communities found this rewarding but required time to undertake key activities to ensure currency and relevance of content. They described adapting their sessions to suit their skill set and inviting experts or community members to co-deliver sessions with them.

Participants suggested some improvements to the training, such as simplifying the content on vaccines and providing more role play demonstrations and examples of how to counter misinformation. While the online webinar format of the Vaccine Champions training sessions allowed them to efficiently and safely reach a large, geographically dispersed audience, it limited opportunities for participants to put the lessons into practice. Training in skills like communication is most effective when it involves a combination of demonstration, observation, practice and feedback [25,26], which could be more easily incorporated in a future face-to-face version of this program.

Formal vaccine champions also requested a forum to connect with other vaccine champions to hear about community concerns, and to hear up-to-date information as they felt their job was not done yet. In the future, linking champions with one another through an online forum or regular meetings would be beneficial to share experiences, workshop responses to common concerns, and maintain motivation among champions.

The concept of utilizing community vaccine advocates is not new, but it is rarely used in isolation so there is little direct evidence of its effectiveness on increasing vaccine uptake. In Victoria, the Vaccine Champions sessions were a key part of a broader community engagement approach which resulted in Victoria achieving a particularly equitable roll out of COVID-19 vaccines [27]. For example, compared with other states, Victoria saw little variation between local government areas, metro and regional locations, or by socio-economic levels. The vaccine uptake amongst the Aboriginal and Torres Strait Islander population was higher in Victoria than any other state at January 2022 [28]. In addition to contributing to vaccine uptake, the Vaccine Champions program was instrumental in supporting CALD community champions to share information and combat misinformation, such as in the Syrian and Iraqi communities [28].

Much of the literature that has been published on community engagement strategies to promote other vaccines comes from low or middle income countries (e.g., community mobilization) [13,29,30], or focuses on a single self-identified community in a high income country (e.g., parents who live an ‘alternative lifestyle’) [12]. This could be because people in high income countries are less likely to view themselves as part of a specific ‘community’, unless they share a defining feature like language or religion. However, anyone with shared values or trusted information sources can be considered a ‘community’ that may be influenced by a vaccine champion. The success of the Vaccine Champions program suggests that high income countries should consider more community-based approaches for vaccine promotion.

A population-wide Vaccine Champions program may be time and resource-intensive, as noted by some participants in our evaluation, which may be why community engagement strategies are most commonly described in the context of mass vaccination campaigns (e.g., polio) [31]. However, as we move away from the campaign nature of COVID-19 vaccination, the program can be adapted and targeted to address routine vaccination as well. For example, the Vaccine Champions program is now expanding internationally with the support and partnership of UNICEF, the Australian Department of Foreign Affairs and Trade (DFAT), and the Australian Regional Immunisation Alliance (ARIA). This regional program involves working with local and national stakeholders in the IndoPacific region to adapt and co-design the Vaccine Champions program for local priority groups to target barriers to COVID-19 and routine childhood vaccination. Feedback from our evaluation has been incorporated into this next phase of the Vaccine Champions program. Key changes include simplifying the content, delivering the training face-to-face, extending it to run for 1–2 days and incorporating many and varied opportunities to practice communication skills and receive feedback. Champions are also followed up and supported to design, plan and deliver their community sessions, including through a peer network of other champions, and are provided with supporting resources.

Our evaluation study had a number of limitations, including primarily that we were only able to evaluate a subset of 20 sessions, rather than the whole Vaccine Champions program. While we collected some basic feedback data on earlier sessions, the rapid and evolving nature of the vaccine rollout meant that we were unable to establish an appropriate and comprehensive process for data collection until 2022. We delivered more sessions in 2021 and focused more on adult vaccination, while the 2022 sessions focused more on the COVID-19 vaccine for children aged 5–11 years. Within our subset evaluation, the survey response rate was only approximately 25%. The sample of twelve qualitative participants was also relatively small, but it did include participants who were formal champions in both 2021 and 2022. It is possible that those who provided feedback were more engaged, supportive or satisfied with the program, so their responses may not be representative of the broader population of those who attended sessions.

## 5. Conclusions

High priority communities who face unique challenges to vaccination can benefit from strong community engagement practices that are community centered and led and build trust and confidence in vaccines [32]. This is particularly relevant to the strong influence that social norms play in supporting vaccination. Continuing to reach these communities and advocate for the importance of vaccination is important. This has been particularly important to increase vaccine uptake for younger age groups (e.g., primary and preschool children, especially those at higher risk of COVID-19) and to provide boosters to priority adult groups.

The Vaccine Champions training program aimed to upskill community, faith and industry leaders belonging to diverse communities in Victoria, to become vaccine advocates in their community. Findings from this study indicate the Vaccine Champions training program was an overall success with broad impact and promising implications for diverse and low resource settings.

It is critical that established community partnerships are maintained and that ongoing support for formal vaccine champions is provided so they can continue to effectively reach their communities and advocate for vaccines beyond COVID-19.

## Figures and Tables

**Table 1:** Characteristics of interview participants.

Participant	Gender	Role/s	Formal Champion?
01	Female	Nurse	No
02	Female	Pharmacist	Yes
03	Female	Community health/engagement officer	Yes
04	Female	Community health/engagement officer	No
05	Female	Community health/engagement officer	No
06	Female	Disability organization representative	Yes
07	Male	General practitioner	Yes
08	Female	Multicultural group representative	No
09	Female	Multicultural group representative	Yes
10	Female	Community health/engagement officer	Yes
11	Male	Community health/engagement officer and multicultural group representative	Yes
12	Female	General practitioner	Yes

**Table 2:** Confidence and satisfaction.

Measure	N	%
**How confident are you in your ability to talk about the risks and benefits of COVID-19 vaccines?**		
More confident	118	94%
About the same	7	6%
Less confident	0	0%
*Missing*	0	
**How confident are you in your ability to find appropriate resources on COVID-19 vaccination information?**		
More confident	112	90%
About the same	12	10%
Less confident	1	1%
*Missing*	0	
**How likely are you to initiate a conversation about COVID-19 vaccines with a person who might be hesitant?**		
Very likely/likely	116	94%
Neutral	6	5%
Not very likely/not likely at all	1	1%
*Missing*	2	
Please rate how satisfied you were with the following:		
**The information about vaccine safety and effectiveness**		
Satisfied/very satisfied	123	99%
Neither satisfied nor dissatisfied	1	1%
Not at all/not very satisfied	0	0%
*Missing*	1	
**The strategies for discussing vaccines with a hesitant person**		
Satisfied/very satisfied	121	97%
Neither satisfied nor dissatisfied	2	2%
Not at all/not very satisfied	2	2%
*Missing*	0	
**The example conversations (role play)**		
Satisfied/very satisfied	113	92%
Neither satisfied nor dissatisfied	8	7%
Not at all/not very satisfied	2	2%
*Missing*	2	
**The question and answer sections of the session**		
Satisfied/very satisfied	122	98%
Neither satisfied nor dissatisfied	2	2%
Not at all/not very satisfied	0	0%
*Missing*	1	
**The quality of the presenters**		
Satisfied/very satisfied	123	100%
Neither satisfied nor dissatisfied	0	0%
Not at all/not very satisfied	0	0%
*Missing*	2	
**How did you find the length of the session?**		
Too short	1	1%
About the right length	122	98%
Too long	2	2%
*Missing*	0	

## Data Availability

Restrictions apply to the availability of these data. Data are available from the authors upon request and with the permission of the Victorian Department of Health. For more information about the training process and materials themselves, the authors welcome enquiries from interested organizations or individuals.

## Appendix A. Survey Instrument

**Table A1:** Vaccine Champions Evaluation Survey.

Question	Response Options
Comparing your level of confidence before and after attending the session:	
How confident are you in your ability to find appropriate resources on COVID-19 vaccination information?	More confident About the same level of confidence Less confident
How confident are you in your ability to talk about the risks and benefits of COVID-19 vaccines?	More confident About the same level of confidence Less confident
How confident are you in your ability to answer difficult questions about COVID-19 vaccines?	More confident About the same level of confidence Less confident
How likely are you to initiate a conversation about COVID-19 vaccines with a person who might be hesitant?	Very likely Likely Neutral Not very likely Not likely at all
Please rate how satisfied you were with the following: The information about vaccine safety and effectiveness	Very satisfied Satisfied Neutral Not very satisfied Not satisfied at all
The strategies for discussing vaccines with a hesitant person	Very satisfied Satisfied Neutral Not very satisfied Not satisfied at all
The example conversation/s (role play)	Very satisfied Satisfied Neutral Not very satisfied Not satisfied at all
The question and answer sections of the session	Very satisfied Satisfied Neutral Not very satisfied Not satisfied at all
The quality of the presenters	Very satisfied Satisfied Neutral Not very satisfied Not satisfied at all
How did you find the length of the session?	Too short About the right length Too long
Which best describes the group or network through which you attended this training?	Victorian government department Please specify (optional): __ Culturally and linguistically diverse community group Aboriginal or Torres Strait Islander community group Industry group or union Healthcare provider group Education provider group Other: ___

**Table A2:** Group Interview Guide (Vaccine Champions or Attendees). Prior to interview commencement: Acknowledgement of country; Study explanation, housekeeping; Consent.

In General:
To do a quick round of introductions: tell me your first name, in what capacity you attended the session (e.g., a health care worker), roughly when you attended the session, and if you went on to deliver any sessions yourself?
**Thinking about the initial session?**
What community group/session did you attend? (both) *Specific to health workers, general?* *Did you go to more than one session?* *Did you set up any other sessions afterwards?*
How did you find the session? (both) *What did you like?* *What could be improved?* *How did you find the presenter?* *Was there any information you wanted to know more about that wasn’t included? * *Did it meet your expectations? Did it give you what you needed?*
What did you think about the content? (both) *Was it pitched at the right level? Was it too complicated or too simple?* *What did you think about the information on vaccines?* *What did you think about the information about supporting vaccination and having discussions?* *What did you like? What could be improved?*
What did you think about the format? (both) *What about the slides?* *If it was held in person, would it have been different? How so?* *Did you refer back to the slides afterwards?* That was all the questions I had about the session delivered by MCRI specifically.
**Champions–delivering sessions and skills learnt**
If you signed up to be a champion, what was your understanding of what would be involved? (champions) *What made you decide to do it?*
How did you find delivering your own sessions? (champions) *What went well? What could be improved?* *Did you end up delivering a session? How many?* *Which community groups did you deliver the sessions to?* *If delivered a number of sessions-did they change over time?*
Can you give an example of how the training session prepared you to deliver the program? Or how it didn’t prepare you? (champions)
Tell me about the materials? (champions) *Did you have to adapt the session to work for you? Describe any changes you made? * *What materials could have made it better? Simpler slides? Flip charts? Role play? Lesson plan?* *How did you find the process of getting materials from Department?* *What did you like? What could be improved?*
When talking about the practically of it all: (champions) *How did the department book sessions for you? * *Were the sessions well organised? * *Who was there?* *Did you have any say over presenting?*
**Using skills**
Tell me about any occasions, where you have applied the skills, strategies, or knowledge you learned from the training? (both) [Outside of the community sessions] *If not, why do you think that is? Is there anything stopping you?* *Tell me an example of when you spoke to someone, or used skills, or changed someone’s mind.*
What was most impactful thing you were involved in, from your perspective? (both) *What made the biggest bang for buck? * *Was it having big sessions? One on one conversations?*
**Future**
What aspects of this process should we carry forward? Or will you carry forward? (both) *What do you hope to take forward?* *Would you use skills to talk about other vaccines? * *Benefits to future community sessions? Would you organise your own session if DH don’t?* *Should we continue to train people to talk to vaccine as we move out of covid/not? * *What is the legacy of this work? For yourself/for the department*?
Is there anything else you would like to tell me, about the Vaccine Champions Training Session, that we haven’t covered? (both)
